# Segmentation of Neuronal Structures Using SARSA (λ)-Based Boundary Amendment with Reinforced Gradient-Descent Curve Shape Fitting

**DOI:** 10.1371/journal.pone.0090873

**Published:** 2014-03-13

**Authors:** Fei Zhu, Quan Liu, Yuchen Fu, Bairong Shen

**Affiliations:** 1 Center for Systems Biology, Soochow University, Suzhou, Jiangsu, China; 2 School of Computer Science and Technology, Soochow University, Suzhou, Jiangsu, China; University of Michigan, United States of America

## Abstract

The segmentation of structures in electron microscopy (EM) images is very important for neurobiological research. The low resolution neuronal EM images contain noise and generally few features are available for segmentation, therefore application of the conventional approaches to identify the neuron structure from EM images is not successful. We therefore present a multi-scale fused structure boundary detection algorithm in this study. In the algorithm, we generate an EM image Gaussian pyramid first, then at each level of the pyramid, we utilize Laplacian of Gaussian function (LoG) to attain structure boundary, we finally assemble the detected boundaries by using fusion algorithm to attain a combined neuron structure image. Since the obtained neuron structures usually have gaps, we put forward a reinforcement learning-based boundary amendment method to connect the gaps in the detected boundaries. We use a SARSA (λ)-based curve traveling and amendment approach derived from reinforcement learning to repair the incomplete curves. Using this algorithm, a moving point starts from one end of the incomplete curve and walks through the image where the decisions are supervised by the approximated curve model, with the aim of minimizing the connection cost until the gap is closed. Our approach provided stable and efficient structure segmentation. The test results using 30 EM images from ISBI 2012 indicated that both of our approaches, i.e., with or without boundary amendment, performed better than six conventional boundary detection approaches. In particular, after amendment, the Rand error and warping error, which are the most important performance measurements during structure segmentation, were reduced to very low values. The comparison with the benchmark method of ISBI 2012 and the recent developed methods also indicates that our method performs better for the accurate identification of substructures in EM images and therefore useful for the identification of imaging features related to brain diseases.

## Introduction

The brain is the center of the nervous system in all vertebrates and most invertebrate animals [1], and it comprises a vast number of interconnected neurons, which are the basic building blocks of the nervous system. Each part of a neuron plays an important role in the communication of information throughout the body [2]–[5]. The connections between neurons in the brain, i.e., synapses, allow neurons to pass signals to individual target cells [4]. The precise patterns of these synaptic contacts are fundamental for neurobiological research [6]. However, synaptic contacts are very small, so they can only be detected using high-resolution electron microscopy (EM).

To produce connectomes based on EM, we need to identify each synapse and trace the axons and dendrites in the brain using images, which technologically is an image segmentation task with the goal of segmenting neuronal structures. Image segmentation, the key step of image processing and image analysis, is the process of dividing the image into several specified areas which have distinct properties for extracting interested objects. The result of neuronal structure segmentation that interests us is the boundary of the neuronal structure [7], [8]. The neuronal structure segmentation task is especially challenging because neurons contain many intracellular organelles such as mitochondria and endosomes. This internal clutter can be distracting. In addition, the external boundaries between neurites and internal boundaries of the intracellular organelles add to the complexity of detection and make the task even harder [9]. Moreover, neuronal EM images are generally low resolution gray images that contain numerous kinds of noise. In many cases, some useful features such as brightness, color, and texture, could be unavailable in EM gray images. Therefore, the results are usually dissatisfactory when conventional image segmentation approaches are applied to the identification of neuronal structures [10].

Therefore, we propose a novel approach for neuronal structure segmentation. As most neuronal EM images are low resolution with noise, Gaussian filtering [11] is applied initially to remove the noise and we generate an EM image Gaussian pyramid [12]. Next, we use Laplacian of Gaussian (LoG) to identify neuron structure boundaries from the generated multi-scale images. After that we make use of a multi-scale fusion algorithm to combine the results from different layers of the Gaussian pyramid to obtain neuron structure boundaries. However, there are usually many gaps in the detected boundaries reducing the quality of identification and adding confusion to next step analysis. Hereby removing gaps is necessary and helpful for us to analyze the EM images. Meanwhile, the uncertainty and unpredictable features of EM gray images make it difficult to prepare a model for missing curving fitting in advance. Thus, we propose a reinforced gradient-descent shaping fitting method to approximate the missing part of the curve. This approach uses the existing curve as an input and adjusts the behavior of an agent when interacting with the environment, which avoids the limitations of the training-and-modeling approach used by most conventional machine learning approaches. Next, we utilize a SARSA (λ)-based curve gap amendment algorithm by using the approximate curve. In the algorithm, the moving point starts at the one end of the incomplete curve and walks through the image and the decisions made by the agent aim to minimize the connection cost, until a closed curve is formed.

### Related Work

Various operators are commonly used for image segmentation, such as Sobel [13], [14], Prewitt [13], [14], Laplace of Gaussian function (or Laplacian of Gaussian function: LoG) [15], Canny [16], Kirsch [13], [14], and Roberts Cross operators [13], [14]. The Sobel operator is a simple and effective tool for image segmentation. However, it is not based on gray image processing, so it cannot separate foreground objects from the background of an image, which may make the outline of an image unsatisfactory. The Prewitt operator computes an approximation of the gradient of the image intensity function. The Prewitt operator tends to lose small amplitude boundary points, which lead to some errors. The LoG operator is used frequently in digital image processing for segmentation and binarization. The LoG operator starts by smoothing the original image suppress noise, before detecting the boundary.

The parameters of the Canny operator can be adjusted to specific requirements to identify boundaries with different characteristics. However, the Canny operator is slightly slow during real-time image processing. The Kirsch operator is a nonlinear detector that finds the maximum boundary strength in a few predetermined directions. The Roberts Cross operator is used for image segmentation and it highlight changes in intensity in a diagonal direction. This operator is simple, but it suffers greatly from sensitivity to noise.

Recent studies have shown that machine learning can improve the accuracy when detecting object boundaries in images. Many features associated with boundaries, such as the brightness, color, gray level, and texture, can be utilized [17]. Dollar et al. [18] proposed a supervised learning algorithm for object boundary detection, which selects and combines features at different scales. Some researchers have aimed to improve optimization constraints, e.g., the metric proposed by Jain et al. [10] aimed to minimize topological disagreements rather than disagreements over boundary location and their approach improved the segmentation performance significantly. In addition, the gap elimination approach proposed by Denk et al. [19] can amend an incomplete curve.

Image segmentation related techniques have been applied widely to biological and biomedical images. Accurate and automatic particle detection by EM is very important for the high-resolution reconstruction of large macromolecular structures. Cardona et al. [9] introduced an approach to the comprehensive anatomical reconstruction of neuronal microcircuitry based on transmission EM sections of a small brain, i.e., the early larval brain of *Drosophila melanogaster*. Yu et al. [20] proposed a method for particle picking based on shape feature detection. Jurrus et al. [21] used Kalman-snakes and optical flow computation to track axons across large distances in volumes acquired by serial block-face scanning EM. Zhang et al. [22] proposed a multi-domain fast-marching method with manual or fit-based multi-seeding to segment meaningful substructures. The dual point decision process developed by Giuly et al. [23] can segment the three-dimensional (3D) structures obtained by 3D microscopy. Seghier et al. [24] described a method for microbleed detection using automated image segmentation, which delivered fairly consistent results compared with the manual method. Plaza et al. [25] aimed to minimize the manual correction and segmentation time by proposing a probabilistic method for reducing manual correction, but without losing the segmentation quality.

Brain magnetic resonance image (MRI)-related information processing is a hotpot for researchers. Gousias et al. [26] segmented neonatal and developed brain MRIs into 50 anatomical regions. Their proposed approach could automatically classify the images into predefined categories, which allowed age-specific brain atlases of neonates to be produced. Yu et al. [27] evaluated the effects of neonatal intensive care and predicted the neurodevelopmental outcomes of high risk newborns using a combination of manual and automated segmentation tools. Wang et al. [28] also argued the importance of infant brain MRI segmentation when quantifying patterns during early brain development. They proposed a longitudinally guided level set method for segmenting serial infant brain MRIs. Keihaninejad et al. [29] utilized a kernel-based class separability criterion to segment structures in brain images, before using a support vector machine to categorize the results. Attique et al. [30] identified tissues using MRIs. Caskey et al. [31] proposed an open-source software tool for tissue segmentation in images. Eggert et al. [32] analyzed and discussed several factors that may affect MRI segmentation in terms of the final segmentation quality and specific adequate performance criteria.

## Metrics Used for Evaluation

### Precision, Recall Rate, and F-score

Many metrics have been used to evaluate the performance of image segmentation task where the goal is to detect boundary. The precision, recall rate, and F-score are used frequently. The precision [33] is the probability that a resulting boundary pixel is a true boundary pixel. The recall rate [34] is the probability that a true boundary pixel is detected. The F-score [35], which is defined for all points on the precision-recall curve, is the harmonic mean of the precision and the recall rate. Definitions of the precision, recall rate, and F-score are as follows.
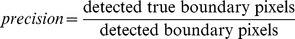
(1)

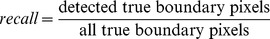
(2)

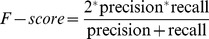
(3)


### Pixel Error

The pixel error [10] of the test labeling of an image relative to the standard labeling is based on the number of pixel locations where the two labeling systems disagree. The pixel error is defined as 1 - the maximal F-score of the pixel similarity, as follows:
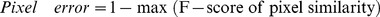
(4)


### Rand Index

The Rand index [36] is a measure of the similarity between two data groups. It can also be used to evaluate the performance of image segmentation. Given an image *S* with *n* pixels, as well as two segmentations *X* and *Y*, let:

*a* be the number of pixels in *S* that are both the same in two segmentations;

*b* be the number of pairs in *S* that are both different in the two segmentations.

The Rand index [36] is then defined as:
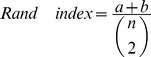
(5)


Rand error, used as a measure of disagreement, is the frequency with which the two segmentations disagree over whether a pair of pixels belongs to the same objects or not.
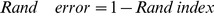
(6)


### Warping Error

There is a type of disagreement when the sketch of an object has been identified roughly whereas the detected boundaries are incomplete. The pixel error metric is overly sensitive to minor displacements in the location of a boundary, which leads to large quantitative differences in the pixel error, although there are no qualitative differences in the interpretation of the image. This shows that the pixel error and Rand error are not good choices in such cases. Thus, we use the warping error [10] to evaluate this type of disagreement. The warping error penalizes the topological disagreements produced when an object splits and merges. Given the pixel error of *T* relative to warpings of L*, the warping error between some candidate labeling *T* and a reference labeling L* can be regarded as the Hamming distance between L* and the best warping of L* onto *T*, as follows [10]:
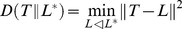
(7)


### Macro Metrics

The aforementioned error rates can be only used to evaluate the performance of a single test. If we want to measure the global performance of a system, we need to use a macro averaging evaluation rate, such as macro precision, which is computed by averaging the labeled precision. Definition of the macro precision rate, macro recall rate, macro F-score, macro Rand error, and macro warping error are as follows:
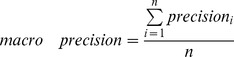
(8)

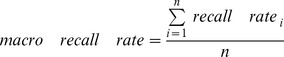
(9)

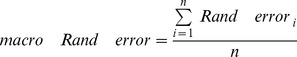
(10)

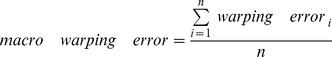
(11)


## Methods

### Criteria for Structure Detection

In general, a good structure detection algorithm should determine the precise location of the boundary. Thus, we propose five criteria that our structure detection algorithm should follow.

#### Rule 1

The approach should minimize the probability of missing an actual boundary point and the probability of mislabeled a non-boundary point.

#### Rule 2

Detected pixels should be located as much as possible in the actual centers of the boundary points.

#### Rule 3

A point is either a boundary point or a non- boundary point, but never both.

#### Rule 4

With topological disagreements, minor differences in the boundary location can be tolerated.

#### Rule 5

The intensity of boundaries should correspond as closely as possible to what a human would perceive.

Based on rule 1, we can obtain the maximal signal-to-noise ratio because the two probabilities in rule 1 are proportional to the opposite directions of the signal-to-noise ratio of the resulting image. To satisfy rule 3, we need to ensure that the boundaries produced are well defined. Therefore, for a given boundary, the point has only one unique result rather than different boundary extraction results in a single curve. If there are different extracted results, then some must be incorrectly identified. As images are representative of actual images in the real-world, it is unavoidable to contain noise in images and it is almost impossible to remove all noise that may cause pixel errors. Minor errors in pixels will have little effect on the understanding of structures and wrapping errors are more sensitive to topology analysis, so the minimization of topological disagreement rather than pixel disagreement is the first goal of this task. The proposed algorithms and the amendment algorithm meet these criteria.

### Multi-scale Fused Structure Boundary Detection

Two key problems must be solved to segment connectomes. First, each synapse must be identified. Second, the “wires” of the brain, i.e., its axons and dendrites, must be traced in images. The complexity of neuronal structures and the negative effects of noise make it difficult to segment structures from EM images. Furthermore, the absence of useful features such as brightness, color, and texture, add the difficulty of segmentation.

Many operators, such as Sobel, Prewitt, Kirsch, and Roberts cross, require little computation, but they are sensitive to noise, which makes it difficult to obtain a satisfactory result. LoG and Canny are effective in determining the locations of boundary points, but these two methods require an appropriate scale. At a small scale, operators are sensitive to boundary points and noise, whereas they are stable at a large scale, but they tend to filter out incorrect details. Thus, we utilize a multi-scale method to describe the diversity of structures and to determine the boundary. At a low resolution scale, our approach identifies boundary points rapidly by suppressing noise and detail, before identifying the position of boundary points precisely at high resolution, and it finally tracks the actual position of the boundary points using a coarse-to-fine tracking strategy.

The Laplacian operator is usually used in image segmentation. However, it is very sensitive to noise. A tradeoff between computational complexity and noise reduction is to carry out Gaussian blur before detecting the boundary using Laplacian operator, which is known as LoG. However, LoG is not much effective in the case of EM images with too much noise information. At present, Gaussian pyramid [12] is getting widely used in many fields, such as image processing, computer vision and signal processing. A Gaussian pyramid decomposes an image into a set of images at different scales, each of which provides specific boundary information. Gaussian pyramid is essentially the representation of image at different scales. An image is blurred by Gaussian function and down-sampled to generate a series of images at different scales for later processing. Inspired by the principle of Gaussian blur, we generate a Gaussian pyramid of the image to be processed, then apply LoG to each level of Gaussian pyramid image to detect neuron structure boundary, and finally combine the detected results together to attain the structures.

The Gaussian pyramid technique generates a stack of successive images, where each pixel contains a local average that corresponds to a pixel neighborhood at a lower level of the pyramid. The detected boundaries are different at various scales, the fine details can be detected at small scale whereas details are often missed at a large scale, such as, a ramp boundary can be only detected easily at a small scale. The last step is to assemble the detected boundaries. However, multi-scale boundary fusion does not mean that the boundaries detected at different scales are simply merged together. The operator discriminates responses at different scales, thereby locating the detected boundary point in a different position, which would cause boundary redundancy if we simply combined them. In general, the resulting boundary produced at a large scale, which tends to be more stable and noise-resistant but poor in terms of positioning accuracy, corresponds to the outline of the image. By contrast, the results obtained at a small scale maintain the rich details of the image and a high positioning accuracy, but they are susceptible to noise interference. Thus, we obtain boundaries with different positions at different scales. However, the boundary positions at two adjacent scales are close to each other. For example, the positional difference at two adjacent scales is just one pixel. Thus, multi-scale boundary fusion should be carried out between adjacent scales. The algorithm used for multi-scale fused structure boundary detection is as follows.

**Algorithm 1:** Multi-scale Fused Structure Boundary Detection.

**Input:** *I*, EM image

*l*, the level of Gaussian pyramid

*α*, predefined value for a boundary point

*β*, predefined value for a non-boundary point

*weight*, predefined weight for Gaussian pyramid of image

**Output:** *boundary*, array for detected result where *boundary*(*x*,*y*) = 1 denotes point (*x*,*y*) is a boundary point and *boundary*(*x*,*y*) = 0 denotes point (*x*,*y*) isn’t a boundary point

1: generate an *l* level Gaussian pyramid of image *I* and get *I*_1_…*I_l._*

2: **for** *i* = 1 **to** *l* **do**

3: detect boundary of image *I_i_* and get *boundary_i_*

4: **end for**

5: **for all** point (*x,y*) in *I*

6: *fusion_account ←* 0

7: **for** *i* = 1 **to** *l* **do**

8: **if** point*_i_* (*x,y*) is a structure boundary **then**

9: *fusion_ i_* (*x,y*) ← α_*_ *weight* of level *i*

10: **else**

11: *fusion_ i_* (*x,y*) *← β*

12: **end if**

13: **for** *i* = 1 **to** *l* **do**

14: *fusion_account ← fusion_account*+*fusion_ i_* (*x,y*)

15: **end for**

16: **if** *fusion_account*<*threshold*

17: point (*x,y*) isn’t a boundary

18: *boundary*(*x*,*y*) *←* 0

19: **else**

20: point (*x,y*) is a boundary

21: *boundary*(*x*,*y*) *←* 1

22: **end if**

23: **end for**

24: **return** array *boundary*

### Boundary Amendment

As neuronal EM images are usually of low contrast, of low resolution and with much noise, the structure detection approaches often have incorrect results and leave many gaps in detected boundary curves. The gaps will make it more difficult to determine the outlines of neuronal structures. Connecting incomplete curves improves our comprehension of images. In general, the gaps are not very large and the curves still appear continuous overall. Thus, we can take advantage of the criteria stated earlier, to exploit the continuity of the boundary pixels in the gradient magnitude or gradient direction and to connect the gaps in the detected boundary.

**Figure 1:**
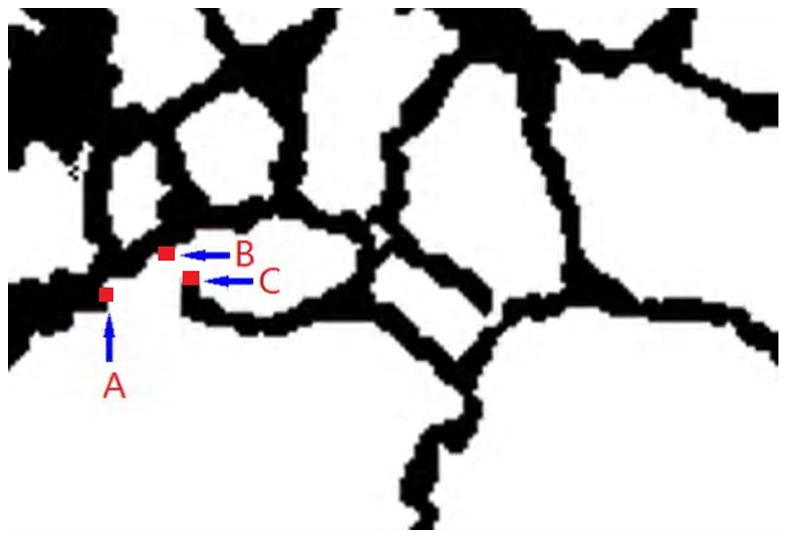
A curve with a gap between A and B rather than A and C which is nearer than B. However connecting A and C is incorrect.

Linking a gap in an incomplete curve can be regarded as connecting the starting point *P*_0_ (*x*_0_, *y*_0_) at one side of the curve with the ending point *P*_1_ (*x*_1_, *y*_1_) at the other side of the curve given some curvilinear trend. It is not appropriate to take the shortest path from point *P*_0_ (*x*_0_, *y*_0_) to the nearest point *P*_i_ (*x*_i_, *y*_i_) as the gap connection curve. As shown in Figure 1, point C is the nearest point to starting point A, but it is not the ending point of gap connection curve. Indeed, point B is the correct point. Connecting point A with point C fails to complete the task of closing the opening curve, but it could also destroy the topology of recognized neuronal structures. Therefore, we propose a SARSA (λ)-based curve shape fitting amendment algorithm to connect gaps in open-ended curves.

### SARSA (λ)

**Figure 2:**
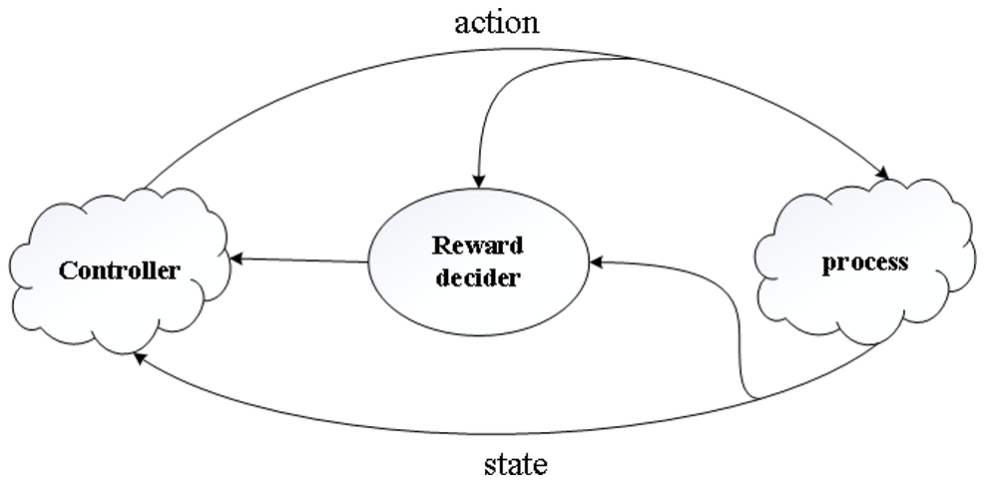
The flow of the interactions in the reinforcement learning framework.

The reinforcement learning [37] problem is considered to be a straightforward framing of the problem of learning from interaction to achieve a goal. The basic reinforcement learning model comprises a set of controllers, process, actions, rewards, and states [38]. The controller obtains the output from a process and applies an action to the process so the behavior of the process can fit predefined requirements. The flow of the interactions during reinforcement learning is shown in Figure 2.

At each time step *t*, the controller selects an action *a_t_* ∈*A* from the action space *A*. As a result, the state changes to *s_t_*_+1_∈*S* from *s_t_*∈*S* according to a transition function *f*: *S*×*A*→[0,∞):
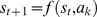
(12)where *s_t_*_+1_ denotes the state at step *t*+1, *s_t_* denotes the state at step *t*, *a_t_* is the action taken at step *t*, and *f* is the transition function.

The controller receives a reward *r_t+_*_1_ according to the reward function 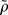:
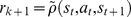
(13)where *r_t+_*_1_ is the received reward by reward function 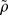 at step *t* with taking action *a_t_* and transferring state from state *s_t_* to state *s_t_*_+1_.

The state-action value function *Q^π^*: *S*×*A*→*R* of some policy *π* yields the return, a long-term reward, from a starting state:
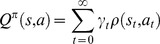
(14)where *Q^π^* is the state-action value function with policy *π*, and *γ*∈[0,1) is the discount rate, which shows how far-sighted the controller is when considering the rewards, and it is also a factor for increasing the uncertainty of future rewards.

Temporal difference (TD) learning [39] is an important concept in reinforcement learning, which is a combination of Monte Carlo and dynamic programming concepts. TD learning occurs according to experience in an environment model. For most real-world applications, TD requires less memory and computational time than conventional approaches, but it usually yields better effects.

SARSA, which is defined by the tuples of state, action, reward, next state, and next action, which is denoted by (*s_t_*, *a_t_*, *r_t+_*_1_, *s_t+_*_1_), is an online TD control method. The update of *Q* [40] depends on (15):
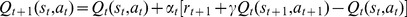
(15)where *Q* is the state-action value function, *γ*∈[0,1) is the discount rate, *a_t_*∈ (0,1] is the learning rate and the *r_t_*_+1_+*γQ_t_*(*s_t_*_+1_, *a_t_*_+1_)-*Q_t_*(*s_t_*, *a_t_*) part is regarded as the TD. The SARSA TD includes the value that *Q* takes in the next state.

SARSA updates according to the difference between the continuous expected reward values, rather than the difference between the expected value and the true value. Therefore, SARSA learning does not need to wait for the end of the task execution.

The TD part of SARSA demonstrates its online characteristic, which means that the data are not necessarily prepared in advance because the system can find out a solution during processing. In an offline approach, the image data should be available in advance because the offline approach needs to know the image model for processing, which is almost impossible in practice because there may be various sophisticated images that make the image model changeable so we cannot predict the image model. Online characteristics of SARSA are superior to offline approaches when solving our problem.

SARSA (λ) is an eligibility trace version of SARSA. Eligibility trace, a memory mechanism used by cognitive science, is associated with backward states. The eligibility trace is updated by:
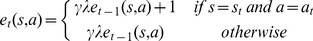
(16)where *e_t_* denotes the eligibility trace at step *t*, *γ*∈[0,1) is the discount rate, *λ*∈ [0,1] is a weight parameter and *Q* is updated by:
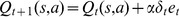
(17)where *δ_t_ = r_t_*_+1_+*γQ_t_*(*s_t_*_+1_, *a_t_*_+1_)-*Q_t_*(*s_t_, a_t_*) is the TD error for state-action prediction.

### Reinforced Gradient-descent Curve Shape Fitting

Curve fitting aims to find out a mathematical expression that models the existing data. The distribution of the points on the curve is restricted by the model. In our study, we consider the curve before the amendment and the curve after amendment when fitting the model. Thus, if we consider the untreated curve and the amended curve as a whole, the continuous portion of the curve can be viewed as one with continuous and dense points whereas the incomplete portion of the curve can be treated as that with discrete and sparse points.

Let the curve be given by:
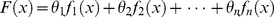
(18)where *θ* is the parameter and *f*(*x*) can be any given basis function. A point on the curve can be represented by (*x*, *F*(*x*)).

Let *F*(*x*) be the real ordinate value of point (*x*, *y*) and 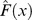 is the estimated ordinate value of point (*x*, *y*). We can see that a lower difference between *F*(*x*) and 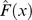 yields a better fitting result. Thus, our goal is to minimize the mean-squared error 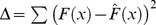. If the derivative of Δ equals zero, 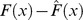 takes the maximum or minimum value of the function. In the present study, 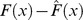 takes the minimum value.

In gradient-descent [41], 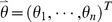 is a parameter vector with real valued components. For each *θ*, we have a representation of a corresponding approximation *Q*, as follows.
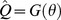
(19)where 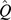 is the approximation value of *Q*, *θ* is the parameter and *G* is the basis function.

Gradient-descent methods minimize the value of the mean-squared error Δ by adjusting the parameter vector gradually with each sampled data:
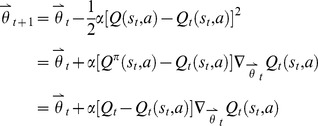
(20)where *α* is the step-size parameter, and *Q_t_*(*s_t_*, *a*) is an approximating function:
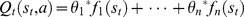
(21)where θ* approximates *θ*. Then, 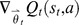 can be represented as 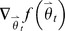, which is the vector of the partial derivatives:
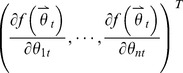
(22)where the derivative vector is the gradient of *f* with respect to 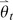.

Various basis functions [42], such as polynomial functions and radial basis functions, have been used widely for image processing, nonlinear approximation, time sequence analysis, data categorization, pattern recognition, information processing, system modeling, and other tasks. Polynomial fitting functions, which aim at fitting a function using a polynomial function, are simple and easy to use. Given the cost of implementation, we selected a polynomial function as the basis function of *f*(*x*). Other types of basis function, such as radial basis functions, trigonometric functions, and 0–1 binary functions, could also be used. A typical polynomial function is defined by:
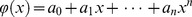
(23)where *a*_0_,*a*_1_,…,*a_n_* are constants.

Therefore, the curve *F*(*x*) is given as follows:
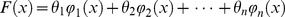
(24)


We put together the items with the same order of *x* and we obtain:
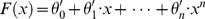
(25)where



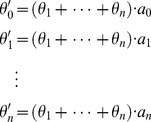
(26)Indeed, equation (25) is a linear basis function version of equation (18). Without loss of generality, we can use equation (27) to represent the curve.
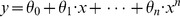
(27)where *θ* is the parameter, and *x* and *y* is the coordinate values of the point.

In our study, the current point position (*x*, *y*) is referred to as the state. The action set is defined as:
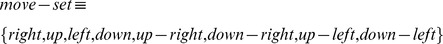



The reward for an action is defined based on the distance between the point with coordinates (*x*, *y*) on the curve and the corresponding approximated point with coordinates (*x’*, *y’*).
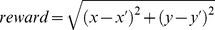
(28)


As the objective can be viewed as minimize total space interval between the approximate curve and real curve, the goal of the proposed SARSA (λ) based algorithm is to minimize the total reward of all points along the curve.

From equation (27), we can see that the reward can be rewritten as follows:
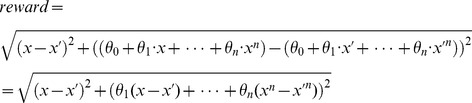
(29)


Let 
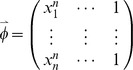
 and 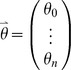, and then *Q* is defined as:
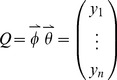
(30)


Correspondingly, the gradient-descent of *Q* is the difference between the two.

The complete algorithm for online reinforced gradient-descent curve shape fitting is given in algorithm 2.

**Algorithm 2:** Reinforced gradient-descent curve shape fitting.

**Input:**the curve *C*, fitting function *F*(*x*), neighborhood Δ, learning rate *α.*

**Output:** parameter vector 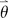


1: 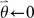


2: 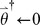


3: get all coordinates of points on the curve in neighborhood Δ

4: **repeat**

5: **for** each point *i* in Δ of the curve *C*

6: *move* ← get move given by *π* for (*x*, *y*)

7: take *move*

8: observe reward *r* by (28) from (*x*, *y*)

9: observe (*x’*, *y’*) for next step

10: observe 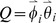


11: step counter ← step counter +1 (batch updating version)

12: **if** reaches predefined step count (batch updating version)

13: 
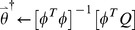


14: step counter ← 0

15: **end if**

16: 
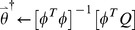
 (instant updating version)

17: 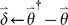


18: 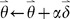


19: (*x*, *y*) ← (*x’*, *y’*)

20: *move* ← *move’*

21: **end for**

22: **until** 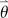 unchanged

23: **return** 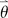


### SARSA (λ)-based Curve Traveling and Amendment

Given a starting point *P*_0_ (*x_0_*, *y_0_*), the ending point *P_n_* (*x_n_*, *y_n_*) could be in any of eight directions, i.e., *up*, *down*, *left*, *right*, *up*-*left*, *down*-*left*, *up*-*right,* and *down*-*right* relative to the point *P*_0_ (*x_0_*, *y_0_*). The neighboring points of *P*_0_ (*x_0_*, *y_0_*) could be on the boundary, which we refer to as a marked point with pixel gray value 1 in the image, or they might not be on the boundary, which we refer to as a blank point with pixel gray value 1 in the image.

If the successive point *P*_1_ (*x*_1_, *y*_1_) of the current point *P*_0_ (*x*_0_, *y*_0_) is a blank point, which indicates that the line that is assumed to fit the curve model should be made up from the current point to the next point to connect them, we will subtract a predefined penalization cost value *gap*, otherwise if the next point is a marked point, we will add a gain to show that the two points have already been connected and the traveling route of the time is correct. Next, the point *P*_1_ (*x*_1_, *y*_1_) is set as the subsequent starting point. We continue exploring until any one of the termination conditions is satisfied, e.g., going back to point *P*_0_ (*x*_0_, *y*_0_), the sum of the penalization cost is less than a predefined threshold, or maximal number of processing episode steps is reached.

In our study, we know little about the model of the structures hidden in complex images. Furthermore, we cannot obtain a complete image beforehand in actual cases. An online approach does not strictly require a model of the environment, reward, and subsequent state probability [40]. Thus, we utilized SARSA (λ) as the basis of our amendment approach.

The slope of a curve is the tangent slope of some point on the curve. In a small domain, a change in the curve slope can be represented as a derivative value of the point, which is the geometric meaning of the derivative. In a small domain, the curve is related to the curve model of some point within its neighborhood. Thus, if the next point is a blank point, we will obtain a model of part of the curve that is the neighborhood of the current point as the expected curve trend. Moreover, it is very difficult to obtain the whole model, whereas it is relatively easy to obtain a model of a small portion of the curve. Therefore, obtaining a curve model of a small area satisfies the characteristics of changing stability and it is also feasible. In our study, when the next point is blank point, we take the current point as the starting point, trace back and move forward within a small neighborhood, obtain a small portion of the curve, approximate a model of the segment, and connect the curve under the supervision of the obtained model.

The current point position (*x*, *y*) is referred to as a state. The action set is defined as:
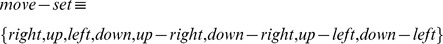



The reward for traveling is given by:
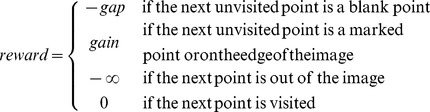
(31)


In algorithm 3, a moving point starts from one end of the incomplete curve and walks randomly through the image where decisions are made by a controller. The point will stop in any of three conditions: (i) returning to the starting point; (ii) the action-value function *Q* is less than the predefined threshold; (iii) the maximum number of processing step in the whole image is reached, which is called an episode step. The SARSA (λ)-based curve traveling and amendment algorithm is as follows.

**Algorithm 3:** SARSA(λ)-based curve traveling and amendment

**Input:**starting point (*x*_0_, *y*_0_), *threshold*, *maximal_step*

**Output:** action-value function *Q* under moving policy

1: initialize *Q*((*x*, *y*), *move*) arbitrarily

2: *episode*_*count* ← 0

3: **for each** point (*x*, *y*) in image **and** *move_i_* in *move-set* **do**

4: e((*x*, *y*),*move_i_*) ← 0

5: **end for**

6: **repeat scanning of image**

7: **for each episode step**

8: initialize (*x_t_*, *y_t_*), *move,* and *e*((*x_t_*, *y_t_*), *move*)

9: *move-set* ←
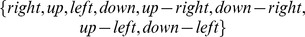


10: take *move* and get new point (*x_t+1_*, *y_t_*_+1_)

11: *r* ← get reward by equation (31)

12: **if** (*x_t_*_+1_, *y_t_*_+1_) is a blank point

13: trace backward neighborhood 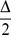 and save points

14: **repeat**

15: move forward to point (*x’*, *y’*)

16: **if** (*x’*, *y’*) is not a blank point

25: 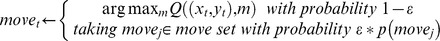


26: *δ* ← *r*+ *γQ*((*x_t_*_+1_, *y_t_*_+1_), *move_t_*_+1_)- *Q*((*x_t_*, *y_t_*), *move*)

27: *e*((*x_t_*, *y_t_*), *move*) ← *e* ((*x_t_*, *y_t_*), *move*)+1

28: **for each** point (*x*, *y*) **in** image, *move_i_* in *move-set* **do**

29: *Q*((*x_t_*, *y_t_*), *move*) ← *Q*((*x_t_*, *y_t_*), *move*)+ *αδe*((*x_t_*, *y_t_*), *move*)

30: *e*((*x_t_*, *y_t_*), *move*) ← *γλe* ((*x_t_*, *y_t_*), *move*)

31: **end for**

32: (*x*, *y*) ← (*x_t_*_+1_, *y_ t_*_+1_)

33 *move_t_*_+1_← *move_t_*

34: *episode*_*count* ← *episode*_*count* +1

35: **until** (*x*, *y*) = (*x*_0_, *y*_0_) **or** *Q*((*x*, *y*), *move*)< threshold **or** *episode_count>*maximal_step

36: **end for**

37: **return** *Q^move^*

## Results and Discussion

There are many competitions in biomedical imaging processing, such as the International Symposium on Biomedical Imaging (ISBI) [43], which focuses on the presentation of technological advances in theoretical and applied biomedical imaging and image computing. ISBI 2012 presented the challenge of automatically segmenting neural structures from EM images. ISBI 2012 provided 30 different sets of serial section Transmission EM (ssTEM) images of the *Drosophila* first instar larval ventral nerve cord (VNC). Each set included a section of ssTEM images that could be used to test the behavior of the approach, as well as corresponding labeled images, which could be used as the evaluation results.

We use the approach that is with boundary amendment and the approach that is without boundary amendment, as well as the Canny, Kirsch, LoG, Prewitt, Roberts Cross, and Sobel operators, to segment structure boundaries from EM images. Figures 3–12 are the 30 test EM images, their corresponding labeled results, and the test results using all eight approaches.

**Figure 3:**
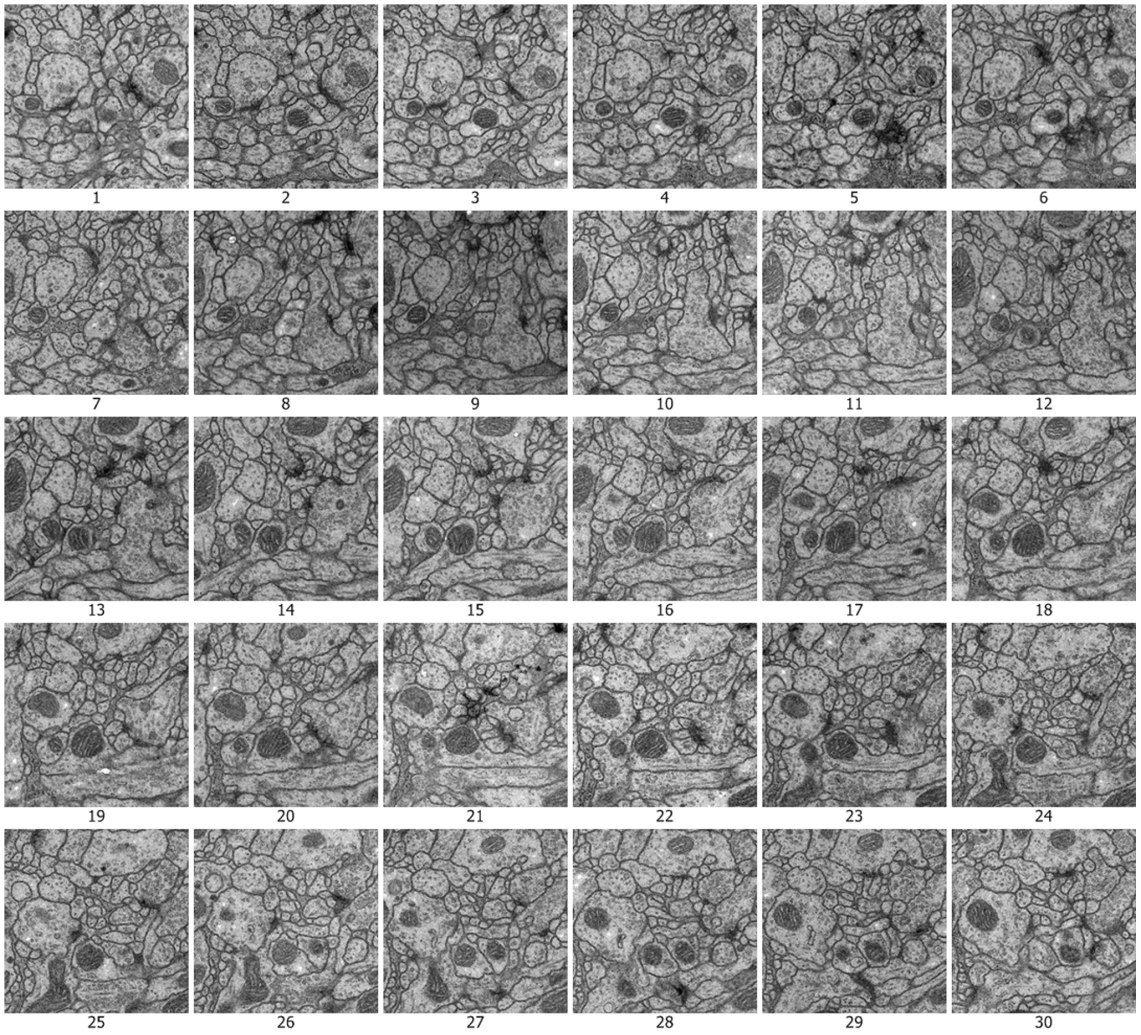
Thirty test EM images for segmentation from ISIB 2012.

**Figure 4:**
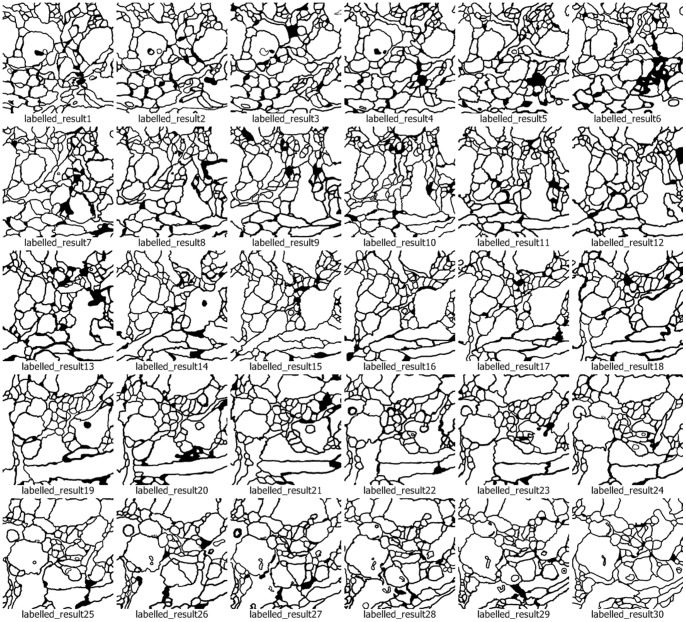
Standard labeled results after neuronal structure segmentation provided by ISIB 2012.

**Figure 5:**
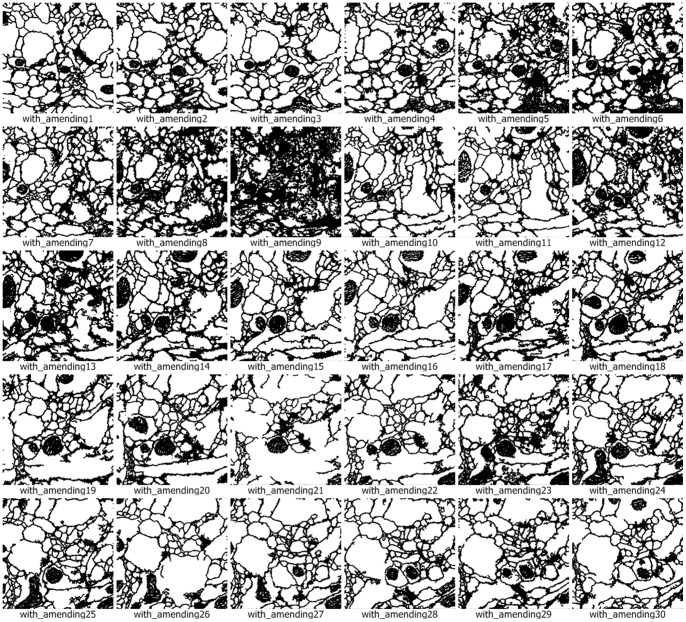
Segmentation results for neuronal structures using our approach after boundary amendment.

**Figure 6:**
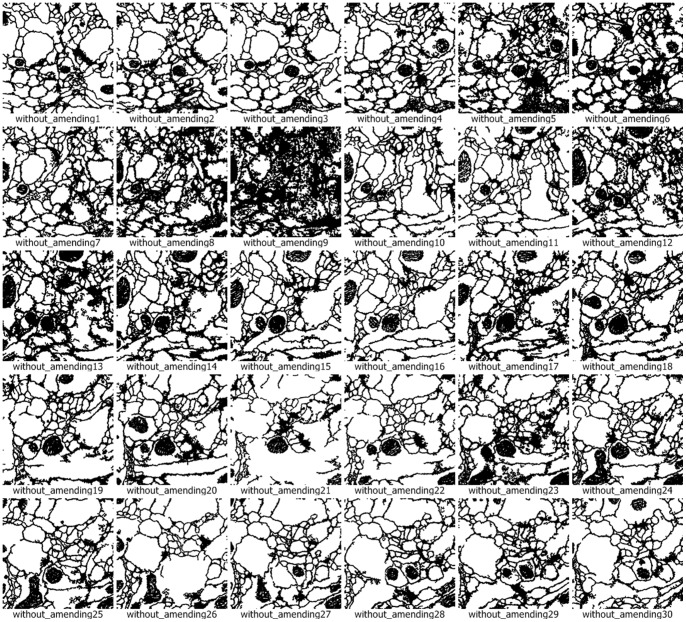
Segmentation results for neuronal structures using our approach without boundary amendment.

**Figure 7:**
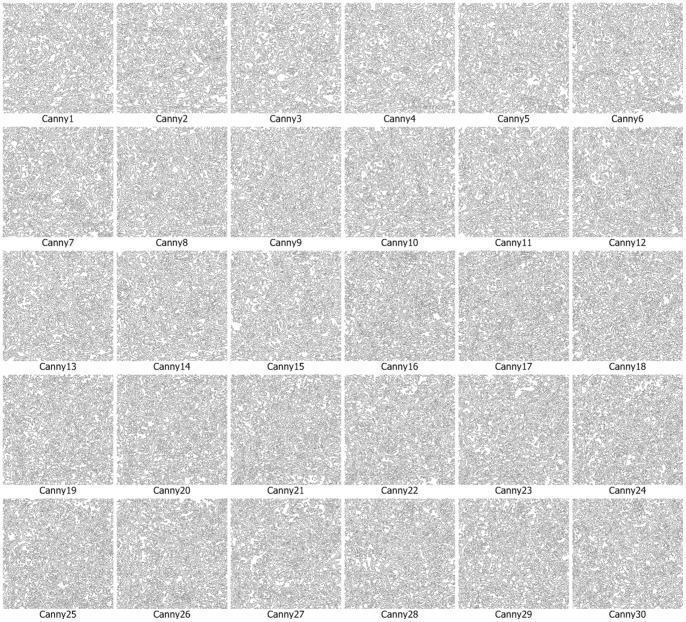
Segmentation results for neuronal structures using the Canny operator.

**Figure 8:**
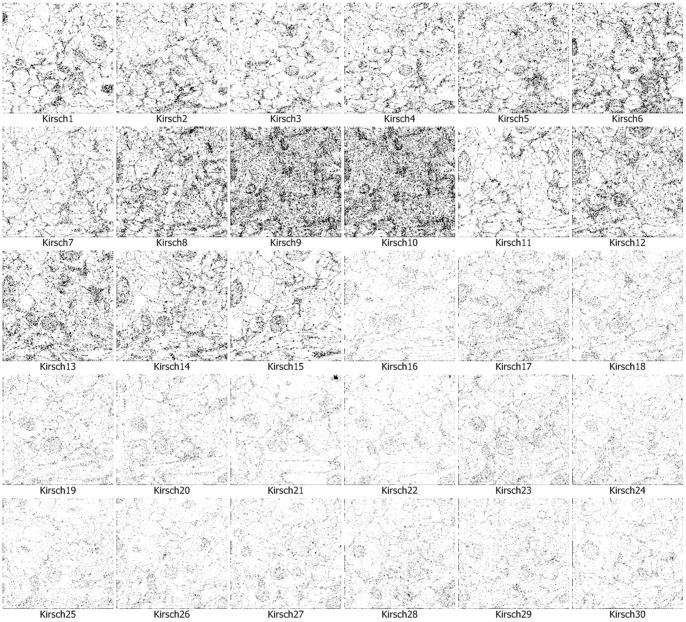
Segmentation results for neuronal structures using the Kirsch operator.

**Figure 9:**
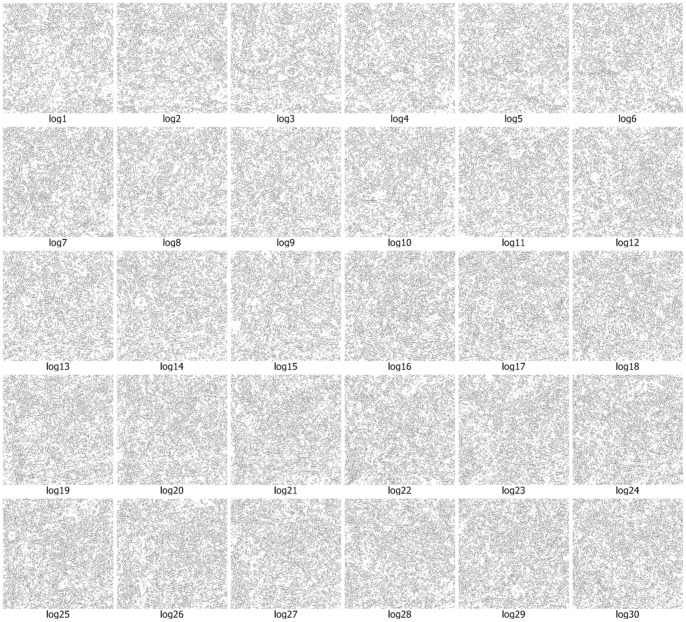
Segmentation results for neuronal structures using the LoG operator.

**Figure 10:**
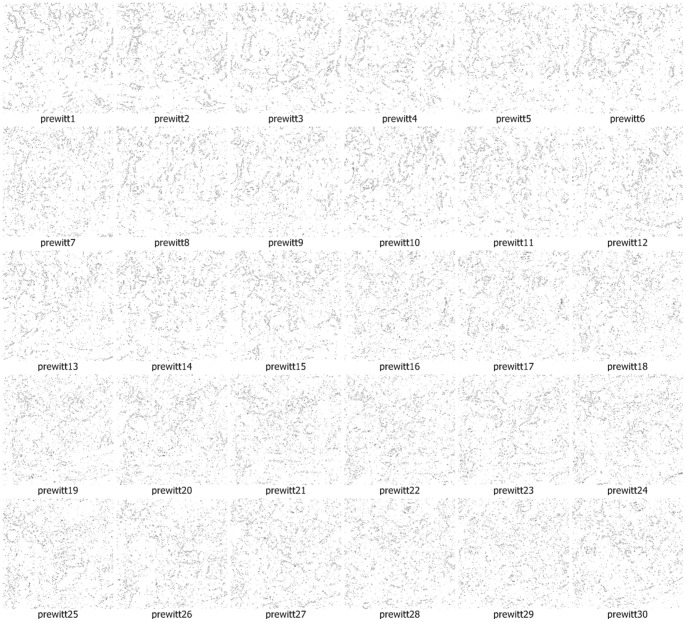
Segmentation results for neuronal structures using the Prewitt operator.

**Figure 11:**
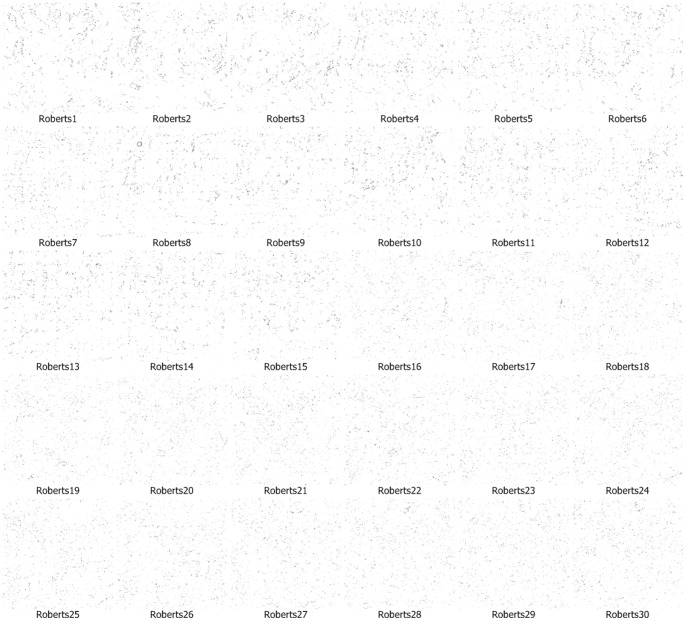
Segmentation results for neuronal structures using the Roberts Cross operator.

**Figure 12:**
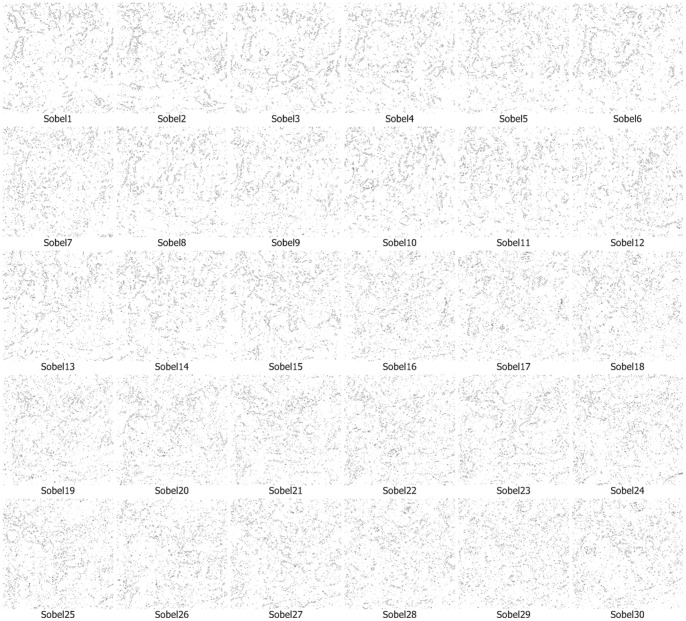
Segmentation results for neuronal structures using the Sobel operator.

We evaluate our results in terms of the pixel error, Rand error, and warping error [10], [44], [45]. Macro averaging evaluation rating results are shown in Table 1. Table 1 shows that both of our methods (with or without amendment) performed better than the other six conventional approaches in terms of all the evaluation indicators. In particular, our approaches performed a better comfortable margin in evaluations with the Rand error indicator and warping error indicator. This is because the conventional approaches simply outline a small part of the structures and they miss the majority of boundaries. Therefore, differences in the Rand error, which evaluates the completeness of structures, are very large.

**Table 1:** Macro averaging evaluation rating results for 30 test EM images from ISBI 2012 using the proposed approach with boundary amendment and the proposed approach without boundary amendment, as well as the Canny, Kirsch, LoG, Prewitt, Roberts Cross, and Sobel operators.

methods	pixel error (%)	rand error (%)	warping error(%)
with_amending	19.36	***3.49***	***0.089***
without_amending	***13.99***	35.78	1.03
Canny	31.76	59.56	1.48
Kirsch	23.79	82.97	2.41
Log	29.52	74.19	2.74
Prewitt	22.69	92.01	1.27
Roberts	22.16	94.78	0.56
Sobel	22.72	91.97	1.31
Total average	23.25	63.12	1.24

A boundary point in an image is a minor portion of the image and most points are non-boundary points, so successfully identifying part of the boundary only leads to a small reduction in the pixel error rate, which compares the image labeling at each location. Thus, although the conventional operators miss the majority of the boundary points, pixel errors of 4/5 conventional approaches were affected only slightly, increasing and falling by 20% to 30%.

By contrast, the Rand error detects the differences between two images, while the warping error measures the disagreement between outlines, thereby providing a relative comparison of the match with the images. The results indicate that both of our approaches worked very well. The main concept used by our amendment approach is to utilize the curve shape and the trend in the image boundary as a supervisor to connect any gaps. The amendments are based on the curve shape and the predicted trend, so it probably causes boundary dislocation in only a very small area. Therefore, the pixel error could even be slightly higher than that before the amendment, as showed in Table 1, whereas the other two evaluation indicators tend to fall as expected. The Rand error and warping error prove to be more useful during boundary segmentation, so we can conclude that the amendment step actually improved identification.

We also compared our boundary amending method with the benchmark method of ISBI 2012, Simple Thresholding [43], [46] which uses a threshold to segment images, and two other newly published methods respectively by Burget [47] and HLFs-RF [48]. The Burget’s method used local-level features to determine segments and their boundaries, and utilized segment-level features to remove the unwanted objects still in the resulting images. In HLFs-RF’s method, thirty four features extracted by traditional way from the pixel, thirty five features extracted by statistical methods from the superpixel, and three context level features among multi superpixels make up the hierarchical level feature vectors for segmentation. Random forest is trained with hierarchical level features to perform segmentation. The results of the above methods are listed in Table 2. Performance measurements by the Rand error and warping error which are the most important for structure segmentation showed our method with boundary amending is best among all the four methods.

**Table 2:** The performance of Simple Thresholding, Burget’s method, HLFs-RF and our method with boundary amending on the ISBI 2012 data set.

methods	pixel error (%)	rand error (%)		warping error(%)		
Simple Thresholding	22.52		44.97		1.714	
Burget’s method	10.23		13.90		0.264	
HLFs-RF	7.913		10.63		0.120	
Our method with boundary amending	19.36		***4.654***		***0.089***	

Table 3 shows the pixel error, Rand error, and warping error values of the two methods: using the approach with amendment and the approach without amendment. These results show that the Rand error and warping error obtained using the approach with amendment were much better than those obtained using the approach without amending, where the difference is an order of magnitude. The pixel error results are not as good as the other two, but we consider that it is better to obtains a complete boundary structure at the cost of a small increase in the degree of the pixel error, as discussed previously, because the Rand error and warping error are more comprehensive indicators, which reflect the quality of the identified boundaries better than the pixel error.

**Table 3:** Pixel error, Rand error, and warping error for 30 EM images from ISBI 2012.

Image#	pixel error (%)		rand error (%)		warping error(%)	
1	14.38	9.64	1.023	18.50	0.068	0.63
2	17.49	11.89	1.091	21.63	0.024	0.99
3	13.62	9.42	0.850	16.58	0.057	0.63
4	16.45	11.81	0.952	22.31	0.074	0.78
5	25.55	20.54	1.710	42.86	0.109	1.33
6	23.89	19.06	1.330	34.54	0.096	1.35
7	16.46	11.87	0.842	21.09	0.089	0.54
8	29.23	22.85	1.993	49.97	0.105	1.93
9	47.76	42.61	3.040	83.16	0.179	2.33
10	14.06	9.59	1.212	22.07	0.073	0.60
11	14.62	9.86	1.366	20.56	0.071	0.56
12	26.56	20.49	2.263	37.56	0.118	1.36
13	24.38	19.35	2.175	35.26	0.097	1.04
14	22.41	16.18	2.266	34.43	0.079	0.98
15	16.82	11.31	1.855	24.16	0.069	0.78
16	15.01	9.92	3.734	37.33	0.098	0.80
17	21.88	15.59	2.585	36.14	0.094	0.96
18	18.59	13.07	1.923	24.34	0.105	0.93
19	16.68	10.97	3.918	37.71	0.088	0.95
20	15.79	10.84	2.187	24.41	0.098	0.91
21	17.67	11.24	59.001	88.23	0.087	1.30
22	16.03	10.38	15.105	67.83	0.085	0.98
23	22.91	16.29	2.319	29.67	0.112	1.20
24	18.53	12.53	2.254	28.00	0.097	1.41
25	20.17	12.81	3.204	33.80	0.103	1.43
26	16.85	10.74	7.942	53.88	0.082	1.07
27	14.92	9.74	1.641	37.41	0.082	0.72
28	14.73	9.78	4.449	34.78	0.083	0.90
29	14.66	9.80	1.916	19.26	0.070	0.70
30	14.77	9.39	3.485	35.88	0.090	0.76
**average**	**19.43**	**13.99**	**4.654**	**35.78**	**0.089**	**1.03**

In each column, the results on the left were obtained using our approach with amendment while those on the right were obtained using the approach without amendment.

The performance of identification on the ISIB 2012 EM images is mostly satisfactory. The proposed method doesn’t work well in few EM images. As shown in Figures 3–12 and Table 3, it could be found that the causes of dissatisfactory identification have three aspects. The first is the low brightness and low contrast of the image, which reduce image segmentation quality and finally lead to low structure identification accuracy. Such as the cases in Figures 3 (5), (8) and (9), the problem can be solved by adopting image enhancement or other image preprocessing methods to increase the image contrast and therefore to improve the segmentation, although the preprocessing need more computational time. The second is the noise occurred in the place where a massive concentration of small neurons locates. The noise will be more affective because the interval between two detected neurons is tiny. In such cases, even a small amount of noise can result in large differences in the final result. For example, in the right part of Figure 3 (12), where multiple small neurons congregate, structure identification performance isn’t good. This kind of misjudgment is hard to be corrected. The last one is related to the predefined threshold in the amending algorithm, *i.e.*, when the length of a gap is larger than the predefined threshold, the algorithm will skip it without any processing. This problem can be resolved by increasing the threshold before amending starts, and this will be more computational intensive.

We find that all three error ratings suggested that optimization actually led to an improvement, which demonstrates that optimization truly amended the curve and enhanced the detection ability. The time cost of the amendment can be ignored because it took just seconds to complete the process in our experiment.

The parameters used in the experiment are shown in Table 4 and Table 5. More episodes (higher episode count) and higher dimensions tended to result in minor improvements in segmentation.

**Table 4:** Parameters used for reinforced gradient-descent curve shape fitting.

parameter	value
episode count	10
boundary pixels ignored	2
maximal amending neighborhood	15
threshold of *θ*	0.001
dimension of *θ*	3
step size *α*	0.1
discounting factor *γ*	0.95

We used a polynomial fitting function as the basis function: y = *θ_n_x^n^*+*θ_n_*_-1_ *x^n^* ^−1^+…+*θ*_0_.

**Table 5:** Some of the parameters used for SARSA(λ)-based curve traveling and amendment.

parameter	value
episode count	1
gap penalty for reward	10
gain for reward	1
step size *α*	0.1

## Conclusions

In this study, we proposed a novel structure boundary detection algorithm where LoG was used at each level of Gaussian image pyramid to attain structure boundaries at different scales. At a larger scale, outlines of neuron structure were extracted and at a smaller scale, the boundary positioning accuracy was improved. A multi-scale fusion algorithm was utilized to combine the detected structure boundaries of different scales together, before a reinforcement learning-based approach was used to fix the gaps in the detected boundaries.

We constructed a model of the curve to detect the trend during the repair of the curve, and a reinforcement learning-based gap fixing algorithm is used to repair the incomplete curve under the supervision of the curve model. The test results obtained using 30 EM images from ISBI 2012 showed that both of our approaches, i.e., with boundary amendment optimization and without boundary amendment optimization, performed better than six conventional approaches. The results also showed that boundary amendment optimization improved the structure segmentation effect.
